# The influence of lower-limb dominance on postural balance

**DOI:** 10.1590/S1516-31802011000600007

**Published:** 2011-12-01

**Authors:** Angélica Castilho Alonso, Guilherme Carlos Brech, Andréia Moraes Bourquin, Julia Maria D’Andréa Greve

**Affiliations:** I MD. Doctoral Student at the Faculdade de Medicina da Universidade de São Paulo (FMUSP), and Researcher in the Laboratory of Kinesiology, Instituto de Ortopedia e Traumatologia, Faculdade de Medicina da Universidade de São Paulo (IOT/FMUSP), São Paulo, Brazil.; II Physiotherapist and Researcher at the Laboratory of Kinesiology, Instituto de Ortopedia e Traumatologia, Faculdade de Medicina da Universidade de São Paulo (IOT-FMUSP), São Paulo, Brazil.; III MD, PhD. Associate Professor in the Department of Orthopedics and Traumatology, Faculdade de Medicina da Universidade de São Paulo (FMUSP), São Paulo Brazil.

**Keywords:** Biomechanics, Proprioception, Postural balance, Posture, Lower extremity, Biomecânica, Propriocepção, Equilíbrio postural, Postura, Extremidade inferior

## Abstract

**CONTEXT AND OBJECTIVE:**

Maintainance of postural balance requires detection of body movements, integration of sensory information in the central nervous system and an appropriate motor response. The purpose of this study was to evaluate whether lower-limb dominance has an influence on postural balance.

**DESIGN AND SETTING:**

This was a cross-sectional study conducted at Faculdade de Medicina da Universidade de São Paulo (FMUSP) and at Hospital do Coração (HCor).

**METHODS:**

Forty healthy sedentary males aged 20 to 40 years, without any injuries, were evaluated. A single-foot balance test was carried out using the Biodex Balance System equipment, comparing the dominant leg with the nondominant leg of the same individual. The instability protocols used were level 8 (more stable) and level 2 (less stable), and three instability indices were calculated: anteroposterior, mediolateral and general.

**RESULTS:**

The volunteers’ mean age was 26 ± 5 years (range: 20-38), mean weight 72.3 ± 11 kg (range: 46-107) and mean height 176 ± 6 cm (range: 169-186). Thirty-four of them (85%) presented right-leg dominance (defined according to which leg they used for kicking) and six (15%) had left-leg dominance. There were no significant differences between the dominant and nondominant legs at the two levels of stability (eight and two), for any of the instability indices (general, anteroposterior and mediolateral).

**CONCLUSION:**

The lower-limb dominance did not influence single-foot balance among sedentary males.

## RESUMO

**CONTEXTO E OBJETIVO:**

A manutenção do equilíbrio postural exige a detecção dos movimentos do corpo, a integração das informações sensoriais no sistema nervoso central e uma resposta motora apropriada. O objetivo deste estudo foi avaliar se a dominância dos membros inferiores influencia o equilíbrio postural.

**TIPO DE ESTUDO E LOCAL:**

Este é um estudo transversal realizado na Faculdade de Medicina da Universidade de São Paulo (FMUSP) e no Hospital do Coração (HCor).

**MÉTODOS:**

Foram avaliados 40 indivíduos sedentários e saudáveis, do sexo masculino de 20 a 40 anos, sem lesões. O teste do equilíbrio unipodal foi realizado no equipamento Biodex Balance System, comparando membro dominante com não dominante do mesmo indivíduo. Os protocolos de instabilidade utilizados foram: nível 8 (mais estável) e nível 2 (menos estável) e três índices de instabilidade foram calculados: anteroposterior, medial/lateral e geral.

**RESULTADOS:**

A idade média foi de 26 ± 5 anos (20-38), massa corporal 72.3 ± 11 kg (46-107) e estatura 176 ± 6 cm (169-186). 34 voluntários (85%) tinham o membro direito como dominante (determinado pelo membro que chuta) e seis (15%), o membro esquerdo. Não houve diferenças significantes entre os membros dominantes e não dominantes nos dois níveis de estabilidade (oito e dois) em nenhum índice de instabilidade (geral, ântero-posterior e medial/lateral).

**CONCLUSÃO:**

A dominância dos membros inferiores não influencia o equilíbrio unipodal em indivíduos sedentários do sexo masculino.

## INTRODUCTION

Balance is defined as the process of maintaining the body's center of gravity within the weight support base. It requires constant adjustment, which is provided by muscle activity and joint positioning.^1-4^

Maintenance of postural balance requires detection of body movements, integration of sensory information in the central nervous system and an appropriate motor response. The body's position in space is determined by visual, vestibular and somatosensory functions. Motor control and dynamic maintenance of balance involve coordinated activity by the muscle kinetic chains.^1,4-6^

The dominant limb can be defined on the basis of muscle strength, functional use and personal preference and these parameters may interfere with balance. Limb dominance should be determined according to which leg the individual chooses and relies on to carry out a variety of functional activities, including maintaining balance.^7^ However, there is a lack of consensus regarding the definition and determinants of lower-limb dominance. The methods most used have been evaluations of kicking and hopping on a single leg.

Comparisons between the limbs are used in orthopedic evaluations and balance tests in order to diagnose functional instabilities, make therapeutic decisions, evaluate results and determine whether patients are in a condition to return to locomotion and/or to sports activities.^8,9^

Unilateral tests are routinely incorporated into clinical practice in order to compare an injured limb with the intact contralateral limb (long jump and high jump tests and single-foot balance test, among others). The contralateral limb serves as a control for the evaluation that is carried out. Postural balance evaluations are one of the ways of measuring proprioceptive loss and they help to determinate the efficiency of habilitation after injury or surgery.^1,8,9^

There has been little investigation relating to variation between the lower limbs and balance, including in relation to dominance.^7^ The body weight support and functional activities of the lower limbs are more important on the dominant side, and thus, dominance might influence postural balance. Unilateral body weight support tests can be used, in order to correlate dominance and postural balance. Our hypothesis was that different lower-limb activities such as body weight support and functional activities with the dominant limb could modify postural balance.

## OBJECTIVES

The aim of the present study was to evaluate whether low-limb dominance has an influence on postural balance.

## METHODS

This study was conducted at the Institute of Orthopedics and Traumatology, Hospital das Clínicas (HC), Faculdade de Medicina da Universidade de São Paulo (FMUSP) and at Hospital do Coração (HCor) after approval granted by the Ethics Committee of Universidade de São Paulo (USP) (number 013/02).

This was a cross-sectional study without intervention, in which 40 male volunteers were evaluated. For the sample size calculation, we assumed the following for the two-tailed hypothesis: alpha value (type 1 error probability) of 5%; beta value (type 2 error probability) of 20%; and thus, a test power of 80% and a difference between the groups regarding the main outcome of 10% To meet these conditions, at least 40 subjects were needed (taking one limb as the unit of analysis, 40 nondominant limbs and another 40 dominant limbs would be needed).^10^

The inclusion criteria were that the volunteers should: a) sign the free and informed consent statement; b) be male; c) be between 20 and 40 years old; d) not have done any physical activity for a minimum of six months; e) not present any neurological, cardiovascular, metabolic, rheumatic or vestibular diseases; f) not have any injuries or previous surgery on the legs; and g) not present any clinical instability in the knees or ankles.

The balance test was carried out using the Biodex Balance System (Biodex, from Biodex Medical Systems, Inc., 2009). The protocols followed the instructions of the equipment manual and previous studies. The instability protocols used were level 8 (more stable) and level 2 (less stable). Level 2 allowed an inclination of up to 20º in the horizontal plane in all directions. Stability varies according to the level of resistance (such that level 8 is the most stable and level 1 is the least stable). Three stability indexes were calculated: anteroposterior stability index, mediolateral stability index and general stability index (sum of the first two).^1,11-15^

### Positioning

**Figure 1:**
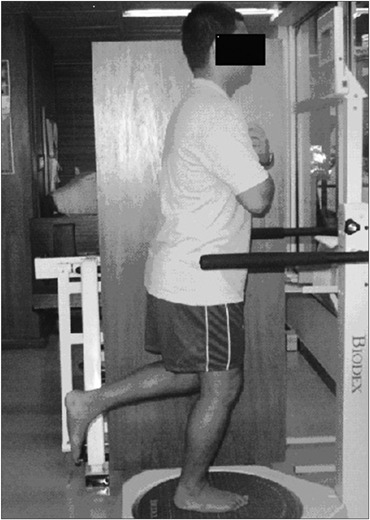
Patient's position in the Biodex Balance System.

The patients were each positioned barefoot on the platform, with their weight supported on one foot and with the corresponding knee semi-flexed at 10º. The contralateral knee remained flexed at 90º. The patients crossed their arms over their chests, looking at the screen in front of them (Figure 1). The platform was released and the patients were instructed to keep themselves in balance with the indicator kept at the center of the target on the screen. When the patient was capable of doing this (i.e. achieving a balance position) without hand support, the foot position was recorded using the platform rail.

### Test

Once the subjects had been positioned, they were instructed not to move their feet until the end of each measurement. The changes were recorded in relation to the center of the platform. Three measurements of 20-second duration separated by one-minute intervals were made on each leg. The result was the arithmetic mean of the three measurements, and this was supplied automatically by the equipment. All the tests started with the dominant leg.

### Statistical analysis

The statistical analysis was performed using SPSS 13.0 for Windows. Differences were taken to be significant when the p value was less than 0.05 (P < 0.05).

The statistical analysis for comparing the dominant leg with the nondominant leg was performed using the paired Student t test for parametric samples.

## RESULTS

The volunteers’ mean age was 26 ± 5 years (range: 20-38), mean weight 72.3 ± 11 kg (range: 46-107) and mean height 176 ± 6 cm (range: 169-186). Thirty-four volunteers (85%) presented right-leg dominance (defined according to which leg they used for kicking) and six (15%) presented left-leg dominance.

Comparing the data through the paired Student t test, there were no significant differences between the dominant and nondominant legs at stability level 8, for any of the instability indices: general P = 0.30, anteroposterior P = 0.73 and mediolateral P = 0.13.

There were no significant differences between the dominant and nondominant legs at stability level 2, for any of the instability indices: general P = 0.27, anteroposterior P = 0.16 and mediolateral P = 0.85 (Table 1).

**Table 1:** Comparison of postural balance between individuals’ dominant and nondominant sides, at stability levels eight and two

	Dominant side Mean (SD)	Nondominant side Mean (SD)	P value
General instability			
Level 8	1.8 (± 0.4)	1.9 (± 0.5)	0.3015
Level 2	4.5 (± 2.1)	4.2 (± 1.8)	0.274
Anteroposterior instability			
Level 8	1.5 (± 0.4)	1.5 (± 0.4)	0.7393
Level 2	3.8 (± 1.8)	3.6 (± 1.5)	0.1629
Mediolateral instability			
Level 8	1.2 (± 0.2)	1.3 (± 0.3)	0.1338
Level 2	2.5 (± 1.0)	2.5 (± 1.1)	0.859
[Paired Student t test]	*P ≤ 0.05		

SD = standard deviation.

## DISCUSSION

The study by Freeman et al.^11^ advocated that activities performed on a single leg should be used in order to decrease the effects of functional instability. Since then, functional activities with the body weight supported on a single foot have been greatly used for evaluating and rehabilitating balance impairments relating to musculoskeletal injuries. However, the difference in performance between the dominant and nondominant limbs needs to be known.

Hoffman et al.^7^ carried out a series of functional tests that they called "functional determination of the dominant limb" and established that the dominant limb was the one that performed the movement with more precision and skill. They confirmed that the dominant leg was the one used for kicking a ball. We used the criterion of the kicking leg to determine dominance in the present study.

As expected, the validity and reliability of balance tests using the Biodex Body System has been considered satisfactory in published studies. For this reason, it was also used in the present study.^12-14^

There was no difference between the dominant and nondominant limb at both levels of instability, but at the level of greater instability of the equipment (level 2), the range of motion was greater because of the need for greater postural adjustments in order to maintain balance.^6,17,18^ However, these findings are similar to those of Alonso et al.,^6^ who found that in groups of sedentary individuals and recreational football players, there was no difference between the limbs. In another study by Alonso et al.^16^ using the same equipment and making the same comparison, but this time on the stability limits among sedentary individuals and judo practitioners, there were no differences between the limbs or in relation to the time taken to achieve effective action in the tests. This was also shown by Tookuni et al.,^2^ who used the Fscan Mat equipment, Hoffman et al.^7^ with a force platform and McCurdy and Langford,^17^ who correlated force and balance by means of squat tests with weights on a platform using single-foot support. These findings are very important because they could be used to evaluate rehabilitation results and postural balance deficit.

Hoffman et al.^7^ stated that asymmetry between the lower limbs was due to acute or chronic lesions and was unrelated to limb dominance. However, McCurdy and Langford^17^ stated that there was no knowledge about the effect of dominance on athletes who used their legs in repetitive asymmetrical activities that would have the potential to generate distinct balance patterns in single-foot evaluations and therefore to interfere with the training and rehabilitation of these athletes.

Our results demonstrate that dominance does not interfere in the evaluation of single-foot balance among healthy sedentary individuals who use their legs for their daily activities and for walking. These findings are particularly important for clinicians and researchers who use comparative evaluations between the limbs, for the purposes of either enabling progression in functional exercises or identifying balance deficits in injured individuals. Further studies are needed with different populations, including elderly people and athletes, in order to confirm these results.

## CONCLUSIONS

In this cross-sectional study, the lower-limb dominance did not influence single-foot postural balance among young sedentary males.

## References

[1] Riemann BL, Guskiewicz KM, Lephart SM, Fu FH (2000). Contribution of the peripheral somatosensory system to balance and postural equilibrium. Proprioception and neuromuscular control in joint stability.

[2] Tookuni KS, Bolliger R, Pereira CAM (2005). Análise comparativa do controle postural de indivíduos com e sem lesão do ligamento cruzado anterior do joelho [Comparative analysis of postural control in individuals with and without lesions on the anterior cruciate ligament of the knee]. Acta Ortop Bras.

[3] Greve J, Alonso A, Bordini AC, Camanho GL (2007). Correlation between body mass index and postural balance. Clinics (São Paulo).

[4] Alonso AC, Vieira PR, Macedo OG, Greve JMD (2007). Avaliação e reeducação proprioceptiva. Tratado medicina de reabilitação.

[5] Voight M, Blackburn T, Ellenbecker TS (2002). Treinamento e testes de propriocepção e equilíbrio após a lesão. Reabilitação dos ligamentos do joelho.

[6] Alonso AC, Greve JM, Camanho GL (2009). Evaluating the center of gravity of dislocations in soccer players with and without reconstruction of the anterior cruciate ligament using a balance platform. Clinics (São Paulo).

[7] Hoffman M, Schrader J, Applegate T, Koceja D (1998). Unilateral postural control of the functionally dominant and nondominant extremities of healthy subjects. J Athl Train.

[8] Kejonen P, Kauranen K, Vanharanta H (2003). The relationship between anthropometric factors and body-balancing movements in postural balance. Arch Phys Med Rehabil.

[9] Davlin CD (2004). Dynamic balance in high level athletes. Percept Mot Skills.

[10] Zar JH (2009). Biostatistical analysis.

[11] Freeman MA, Dean MR, Hanham IW (1965). The etiology and prevention of functional instability of the foot. J Bone Joint Surg Br.

[12] Arnold BL, Schmitz RJ (1998). Examination of balance measures produced by the biodex stability system. J Athl Train.

[13] Hinman MR (2000). Factors affecting reliability of the biodex balance system: a summary of four studies. Journal of Sport Rehabilitation.

[14] Hornyik ML, Harter RA (2001). Reliability of limits of stability testing: a comparison of two dynamic postural stability evaluation devices. Journal of Athletic Training.

[15] Alonso AC, Greve JMA, Macedo OG, Pereira CA, Souza PCM (2003). Avaliação isocinética dos inversores e eversores do tornozelo: estudo comparativo entre atletas de futebol e sedentários normais [Body weight influence on respiratory pressures on sitting supine and upright positions]. Rev Bras Fisioter.

[16] Alonso AC, Bronzatto E, Brech GC, Moscoli F (2008). Estudo comparativo do equilíbrio postural entre atletas de judô e indivíduos sedentários. Brazilian Journal of Biomechanics = Revista Brasileira de Biomecânica.

[17] McCurdy K, Langford G (2006). The relationship between maximum unilateral squat strength and balance in young adult men and women. Journal of Sports Science and Medicine.

[18] Ageberg E, Zätterström R, Moritz U, Fridén T (2001). Influence of supervised and nonsupervised training on postural control after an acute anterior cruciate ligament rupture: a three-year longitudinal prospective study. J Orthop Sports Phys Ther.

